# Cell-Specific Transcriptome Analysis Shows That Adult Pillar and Deiters' Cells Express Genes Encoding Machinery for Specializations of Cochlear Hair Cells

**DOI:** 10.3389/fnmol.2018.00356

**Published:** 2018-10-01

**Authors:** Huizhan Liu, Lei Chen, Kimberlee P. Giffen, Sean T. Stringham, Yi Li, Paul D. Judge, Kirk W. Beisel, David Z. Z. He

**Affiliations:** ^1^Department of Biomedical Sciences, Creighton University School of Medicine, Omaha, NE, United States; ^2^Chongqing Academy of Animal Science, Chongqing, China; ^3^Department of Otorhinolaryngology-Head and Neck Surgery, Beijing Tonren Hospital, Capital Medical University, Beijing, China; ^4^Department of Otolaryngology-Head and Neck Surgery, University of Nebraska Medical Center, Omaha, NE, United States

**Keywords:** hair cell, pillar cell, deiters' cell, transcriptome, RNA-seq, mouse

## Abstract

The mammalian auditory sensory epithelium, the organ of Corti, is composed of hair cells and supporting cells. Hair cells contain specializations in the apical, basolateral and synaptic membranes. These specializations mediate mechanotransduction, electrical and mechanical activities and synaptic transmission. Supporting cells maintain homeostasis of the ionic and chemical environment of the cochlea and contribute to the stiffness of the cochlear partition. While spontaneous proliferation and transdifferentiation of supporting cells are the source of the regenerative response to replace lost hair cells in lower vertebrates, supporting cells in adult mammals no longer retain that capability. An important first step to revealing the basic biological properties of supporting cells is to characterize their cell-type specific transcriptomes. Using RNA-seq, we examined the transcriptomes of 1,000 pillar and 1,000 Deiters' cells, as well as the two types of hair cells, individually collected from adult CBA/J mouse cochleae using a suction pipette technique. Our goal was to determine whether pillar and Deiters' cells, the commonly targeted cells for hair cell replacement, express the genes known for encoding machinery for hair cell specializations in the apical, basolateral, and synaptic membranes. We showed that both pillar and Deiters' cells express these genes, with pillar cells being more similar to hair cells than Deiters' cells. The fact that adult pillar and Deiters' cells express the genes cognate to hair cell specializations provides a strong molecular basis for targeting these cells for mammalian hair cell replacement after hair cells are lost due to damage.

## Introduction

The mammalian auditory sensory epithelium, the organ of Corti, contains hair cells (HCs) and supporting cells (SCs). HCs are sensory receptor cells with the hallmark of the stereocilia bundle in their apical surface. The stereocilia bundle together with the mechanotransduction apparatus are responsible for converting mechanical stimuli into receptor potential in HCs. HCs also contain structural and functional specializations that mediate mechanical property, electrical activities, and synaptic transmission in the basolateral and synaptic membranes (Hudspeth, 2014; Fettiplace, 2017). The SCs, which include inner phalangeal cells, pillar cells, Deiters' cells, Hensen cells, and Claudius cells maintain homeostasis of the ionic and chemical environment of the cochlea as well as contribute to the stiffness of the cochlear partition (Slepecky, 1996; Raphael and Altschuler, 2003).

The HCs are highly vulnerable to damage from exposure to ototoxic drugs and noise. While spontaneous proliferation and transdifferentiation of SCs are the source of the regenerative response to replace lost HCs in lower vertebrates, SCs, such as pillar and Deiters' cells in adult mammals no longer retain that capability (Géléoc and Holt, 2014). In recent years, gene therapy has been used to target adult mammalian SCs for transdifferentiation to replace the lost HCs with some success (Izumikawa et al., 2005; Mizutari et al., 2013). The molecular mechanisms inhibiting spontaneous transdifferentiation of these cells to HCs are not clear. An important first step to revealing their biological properties and potentials to be converted into HCs is to characterize their cell-type specific gene expression profiles (i.e., transcriptomes) and compare their transcriptomes with those of HCs, as such analyses can uncover cell-specific transcripts that underlie biological properties of those cells. Recent studies have examined transcriptomes of several SC subtypes (Burns et al., 2015; Waldhaus et al., 2015) and a mixed population of SCs (Maass et al., 2016; Cheng et al., 2017) as well as HCs (Burns et al., 2015; Cai et al., 2015; Elkon et al., 2015; Scheffer et al., 2015) from embryonic and neonatal mice. However, no cell type-specific transcriptomes of SCs from the adult organ of Corti have been characterized to date. A thorough analysis of the adult SC and HC transcriptomes is important because converting SCs to HCs following onset of acquired deafness would likely happen at the stage when SCs and HCs are already mature. Using RNA-seq and the suction pipette technique that we pioneered (He et al., 2000; Zheng et al., 2000; Liu et al., 2014), we analyzed transcriptomes of 1,000 individually collected pillar and Deiters' cells from adult mice, as well as inner and outer HCs (IHCs and OHCs), the two types of sensory receptor cells. We examined the differences and similarities among these four cell types since pillar and Deiters' cells have been the target for gene therapy to replace lost HCs (Izumikawa et al., 2005; Mizutari et al., 2013). Our main goal is to determine whether adult pillar and Deiters' cells express the genes encoding HC specialization machinery for mechanotransduction, electrical and mechanical activities, and synaptic transmission. The expression of the genes cognate to HC specialization machinery in pillar and Deiters' cells would suggest that they have the intrinsic capacity to function as HCs and that their conversion is dependent upon the activation of the appropriate molecular signals. We also compared transcriptomes between pillar and Deiters' cells to determine which cell type has more robust intrinsic HC-like properties. Our cell-specific transcriptome analyses showed that both pillar and Deiters' cells express the genes encoding machinery for HC specializations, with pillar cells being more similar to HCs than Deiters' cells. The fact that adult SCs express those genes provides a strong molecular basis for targeting these cells for mammalian HC replacement after HCs are lost due to damage or aging.

## Materials and methods

### Cell isolation, identification, and collection

Adult CBA/J mice (28–35 days old) were used for transcriptomic analysis and quantitative PCR. For immunocytochemistry, CBA/J and B6.129S-Atoh1tm4.1Hzo/J mice aged between 1 and 3 months were used. The Atoh1tm4.1Hzo/J targeted reporter mice contained an enhanced green fluorescent protein in Atoh1-expressing cells (such as HCs). To isolate HCs, the auditory sensory epithelium was dissected out and transferred to a small Petri dish containing an enzymatic digestion medium (1 ml L-15 and 1 mg Collagenase IV from Sigma). The tissue was transferred to a small plastic chamber containing enzyme-free culture medium (Leibovitz's L-15, 7.35 pH, 300 mOsm) after 5-min incubation at room temperature (20 ± 2°C). HCs and SCs in the chamber were separated by gentle trituration of the tissue with a 200 μL Eppendorf pipette tip. The chamber was then mounted onto the stage of an inverted Olympus IX71 microscope. Before isolated cells were collected, fresh L-15 medium was used to perfuse the chamber to wash out debris for 5 min.

**Figure 1:**
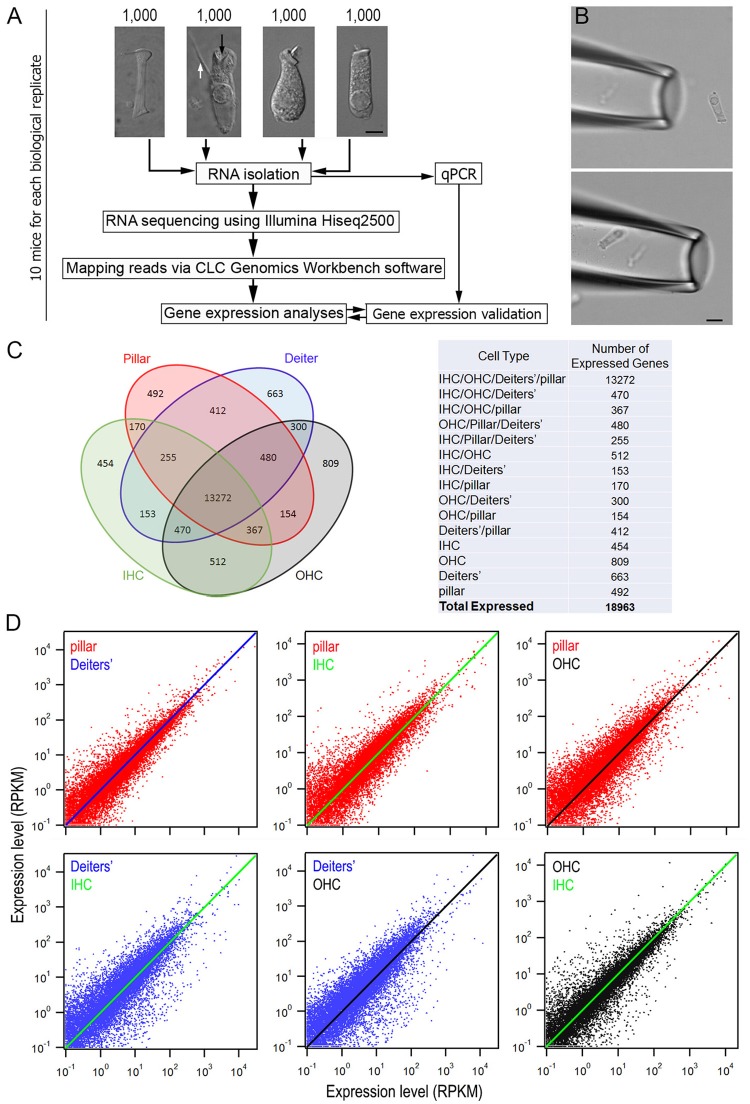
Comparison of gene expression profiles among four different cell types from the adult organ of Corti. **(A)** An overview of the study design for cell collection, RNA preparation and RNA-sequencing. Representative images (from left to right) of an isolated pillar cell, Deiters' cell, IHC, and OHC from adult mice are also presented. Bar: 5 μm. **(B)** A pick-up pipette before an isolated Deiters' cell was drawn into the pipette. Bar: 10 μm. **(C)** Venn diagram depicting the number of expressed genes (RPKM > 0.1) in four cell types. The 18,963 expressed genes were sorted into 1 of 15 categories (listed in the table on the right). The number indicates the total genes shared among two or more cell types or those uniquely expressed by a single cell type. **(D)**: The top three panels present the expression value of pillar cells (red dots) with the reference to that of Deiters' cells (blue line), IHCs (green line) and OHCs (black line), while the bottom two panels (left and middle) show the expression value of Deiters' cells (blue dots) with the reference to that of IHCs (green line) and OHCs (black line). Comparison between OHCs and IHCs is also presented. Only those transcripts whose expression value is equal or greater than 0.1 RPKM are shown.

Pillar cells, Deiters' cells, IHCs and OHCs still retained their distinct morphological features and were easy to be identified after being isolated. Representative images of pillar cell, Deiters' cell, IHC and OHC are presented in Figure 1A. The distinction between IHCs and OHCs after isolation has been described before (He et al., 2000; Liu et al., 2014). The pillar cell possesses a large number of microfilaments and microtubules arranged in bundles along the length of the cell with a footplate in one end and a head portion in another end. The Deiters' cell forms a cup (marked by a black arrow in Figure 1A) that holds the synaptic pole of an OHC. Another important feature of the Deiters' cell is its phalangeal process (marked by a white arrow in Figure 1A), which is an important feature used for identification of Deiters' cells in isolation. Because of these morphological distinctions, these four different cell types were unlikely to be confused for each other. More importantly, we avoided to collecting any cells when their identity was ambiguous. We noted that although the whole basilar membrane and organ of Corti were dissected out for cell isolation and collection, it is likely that most HCs and SCs were from mid to apical turns of the cochlea. This is because HCs and SCs in the basal turn region (especially the far basal end) were more vulnerable to damage and most of them might not survive during isolation. However, we previously measured the length of OHCs in the hemicochlea preparation where the HC condition was better maintained (He et al., 2004; Dallos et al., 2008). We found that the apical turn OHCs from adult mice were ~25 to 30 μm, whereas the basal turn OHCs were ~14 to 18 μm. Some OHCs with the length of ~15 to 17 μm were observed in our preparation. Thus, those cells may come from the basal turn.

The suction pipette technique was used to collect solitary cells (Figure 1B). Detailed information is provided elsewhere (He et al., 2000). Briefly, two pickup (suction) pipettes were prepared from 1.5 mm thin-wall glass tubing pulled by an electrode puller and each pick-up pipette was designated for only one cell type to prevent contamination in the pipette. The diameter of the pipette was ~30 μm. The pipettes, held by two separate electrode holders, were mounted on two micromanipulators (Leitz, Germany). Two micrometer-driven syringes, connected to the suction port of the pipette holders, were used for drawing in the cells or expelling cells. A video showing a mouse OHC being drawing into a pickup pipette is presented as Supplementary Video 1. Solitary cells in the pipette were transferred to a microcentrifuge tube containing 50 μl RNAlater (Thermo Fisher Scientific, Waltham, MA) after 4 to 5 cells were accumulated in the pipette. These steps were repeated until ~100 to 200 pillar cells and Deiters' cells or 80–100 IHCs and 200 OHCs were collected from each animal within an hour. Approximately 10 mice were used for collecting 1,000 pillar cells and 1,000 Deiters' cells, as well as for the same number of IHCs and OHCs. 30 mice were used to prepare three biological replicates for the experiments.

### RNA preparation and RNA-sequencing

An overview of the study design for cell collection, RNA preparation and RNA-sequencing is depicted in Figure 1A. Approximately 1,000 cells of each type were separately suspended in RNALater (~100 μL in total volume) and total RNA, including small RNAs (> ~18 nucleotides), were extracted and purified using the Qiagen RNeasy mini plus Kit (Qiagen, Germantown, MD). To eliminate DNA contamination in the collected RNA, on-column DNase digestion was performed. Total extracted RNA from 1,000 cells ranged from 5 to 10 ng/μl. The quality and quantity of RNA were examined using an Agilent 2100 BioAnalyzer (Agilent, Santa Clara, CA).

Three biological replicates, separately prepared from pillar cells and Deiters' cells, were used to produce genome-wide transcriptome libraries. Libraries were also produced from three biological replicates of OHCs and two biological replicates of IHCs. The SMART-Seq V4 Ultra Low Input RNA kit (Clontech Laboratories, Inc., Mountain View, CA) and the Nextera Library preparation kit (Illumina, Inc., San Diego, CA) were used. An Agilent 2100 Bioanalyzer and a Quibit fluorometer (Invitrogen, Thermo Fisher Scientific) were used to assess library size and concentration prior to sequencing. Transcriptome libraries were sequenced using the HiSeq 2500 Sequencing System (Illumina). Three samples per lane were sequenced, generating ~100 million, 100 bp paired-end reads per sample. The files from the multiplexed RNA-seq samples were demulitplexed and fastq files were obtained.

To individually map the reads (paired-end reads) to the mouse genome (mm10, build name GRCm38), CLC Genomics Workbench software (CLC bio, Waltham, MA, USA) was used. Reads were mapped to exonic, intronic, and intergenic sections of the genome. Gene expression values were normalized as reads per kilobase of transcript per million mapped reads (RPKM). Gene expression estimates were derived from the mapped reads using HTSeq-count (Anders et al., 2015), while DESeq (https://www.basepairtech.com/) was used to identify differentially expressed genes with a false discovery rate (FDR) set to 5% and a fold change threshold of 2 with a minimum normalized read count set to 10% of the median normalized read count (Anders and Huber, 2010). The raw data together with the searchable dataset (in Excel format) are available from the National Center for Biotechnology Information-Gene Expression Omnibus (GEO) (GEO submissions number: GSE111347 and GSE111348). The mean RPKM gene expression values from the replicates of pillar cells, Deiters' cells, IHCs and OHCs are included in Supplementary Table 1.

### Bioinformatic analyses

The differentially expressed genes were examined for enrichment of known biological processes using DAVID (Huang da et al., 2009). Gene clusters tested for enrichment of specific signaling pathways were examined using pathway databases, such as Reactome (https://reactome.org/PathwayBrowser/). Identification of differentially expressed genes was facilitated using Ingenuity IPA program (www.ingenuity.com). Clustering and principal component analysis (PCA) were carried out using the XLStat package (https://www.xlstat.com/en/solutions/base). Hierarchical clustering of gene expression via K-means analysis was conducted with iDEP online bioinformatics platform (http://bioinformatics.sdstate.edu/idep/). Entrez Gene, HGNC, OMIM, and Ensembl database were used for verification, reference, and analyses. Additional resources, such as the gEAR (www.umgear.org) and SHIELD (https://shield.hms.harvard.edu/index.html) were also used for reference and verification.

The differentially expressed genes from each replicate were uploaded into the DAVID bioinformatics suite for gene ontology analysis. Gene Ontology terms related to biological processes were obtained, and REVIGO (Supek et al., 2011) was used to summarize and visualize the long list of gene ontology terms using default parameters. The threshold to detect redundancies used was set arbitrarily to 0.1.

### Quantitative real-time PCR

A custom-made 48 well RT2 qPCR Primer Assay Plate from Qiagen (Germantown, MD) was used for q-PCR verification. The oligonucleotide primers based on AB 7500 Fast Real-Time PCR system (Thermo Fisher Scientific) were generated designed using Qiagen interactive website (https://www.qiagen.com/us/shop/genes-and-pathways/batch-configuration/?type=plate&producttypeid=10). Sequence information for the primers used in our study and qPCR primer assay catalog numbers are provided in Table 1. Forty-one genes that showed differential expression in pillar or Deiters' cells and two genes that did not show differential expression in RNA-seq analysis were included in the RT-qPCR array. Five controls (often used for genomic DNA contamination control (GDC), positive PCR control (PPC), reverse transcription control (RTC) and one housekeeping gene (Sdha) were also included in the plate. We calculated ΔCt values (ΔCt = Ct^(GOI)^ – Ct^AVG HKG^) of each gene (gene of interest or GOI) after normalizing to Ct value of a house-keeping gene (HKG). For comparing differential expression of a gene between pillar and Deiters' cells, we calculated ΔΔCt, where ΔΔCt = ΔCt (pillar) − ΔCt (Deiters'). Thus, a positive value would suggest a higher expression value in pillar than Deiters' cells, whereas a negative value suggests higher expression in Deiters' than in pillar cells. Fifteen additional mice were used to prepare three biological replicates of pillar and Deiters' cells for qPCR validation.

**Table 1:** Sequences of oligonucleotide primers for q-PCR.

	**Forward primer**	**Reverse primer**
Bmp2	GCTTCCGTCCCTTTCATTTCT	AGCCTCCATTTTTGGTAAGGTTT
C1ql1	GGGGCAACAGCAACAAATAC	CCTTGGTCAGGCAATTTGAA
Cd164l2	CCCCATCCCTGAAGACCAC	TGGGGTCAGATTAGTGTCTGG
Chrna9	CGTGTGATCTCCACCAGTGT	TCCTTCATCCCTTTATCCTTGA
Chrna10	GCTCACAAGCTGTTTCGTGAC	ACTTGGTTCCGTTCATCCATATC
Coch	TGGCCTCTAAACCCAAAGAG	ATCCCCTTGCACGTATTCCT
Dnajc5b	ATTTTTGTTGCTGCCTTTGC	AGGCTGGAGAACAACTGGAA
Ednrb	AAAGCCAACGATCACGGATA	CCTTTCTGCTAGCATGGTTTTT
Elmod1	AGAGGATCATAAAACAGCTGC AGAA	CCTAAGCAGAGATCTAGGAT GACA
Etv1	TTAAGTGCAGGCGTCTTCTTC	GGAGGCCATGAAAAGCCAAA
Fcrlb	ACCACCATCTTCAAGGGAGAG	TACCAGAGAGTGCTAATGGGC
Gsn	CCCCATCACAGTCGTTAGGC	CCGGTCCAAAGGATCCAC
Kcne1	ATGAGCCTGCCCAATTCCAC	GAGCTGAGACTTACGAGCCA
Kncn	CATCCTACCTGCCATAGGAAA	CTGCGGGCTCCTTCTTTATT
Lhfpl5	TCCTCTCCTTCCTGGCTTTT	CCTCGGTTGCTTCAGACTTC
Luzp2	GCGACTATACTGGCAAATGCT	GGCCACTTTCAGCACTTTTT
Myo7a	AGGGGGACTATGTATGGATGGA	ATGTGCGTGGCATTCTGAGG
Otof	CTGACACGGCATTCGTCTG	CCTGGGAGGCTGTAAAGGAA
Prdm12	GGAATGAGCAGGAGCAGAAC	TTTCCATACCACACCAGCAG
Scarf2	ATGAGTGTGGGATAGCGGTGT	GGCACTTTGTGTCGCAGTT
Slc1a3	GCACCAAGTGTTGGAAACTG	TTCAAATGTAGGCTAAAACC GATA
Slc7a14	GAGGGAACTGGGACCACCT	TCTGCATAGCACACACCTGATA
Slc17a8	GGAGACAGAACTCAACCACGA	TTCGGCCTGGTAGGATAATG
Slc26a5	CACTCATTATGGGAGCGAGA	TCCGTCTACTTCTGCATCCAC
Srd5a1	GCTTTGCCCTGTGGTTAGTG	CGAGCTCCCCAAAATAGTTG
Tmc1	GAAATGGGCAAAATTCCTCCGA	TGAAGGTCAACACAAAGAG AACC
Tmem54	TTCTGTCTGGCGGAGAGTATG	GGCTTGCTTGTATCGTGTGGT
Dazap2	CAGCCTACCTACCCTGTGC	CATGGGGATTGTGGAGCCTA
Sdha	ACACAGACCTGGTGGAGACC	GGATGGGCTTGGAGTAATCA
Apobr	TGGGCTACATCAGGCTTTGAG	CTCTCCTACAACCTTCCCCTC
Bdh2	CTGCCTGGGTGATCATCTCT	TTGATTTATTTACCACAGCTT CAGA
Bmp4	GGCTGGAATGATTGGATTGT	CACAACAGGCCTTAGGGATA
Clic6	CGACCGGGAGATTGAACAC	ACAGAGGGCTCTCCTGGAA
Cplx1	AAGAAGAAGGAGGAGCGTGA	CATCTTCCTCCTCTGGCTCA
Ctgf	ACTGTCCTGGGGACAATGAC	CTGTAATTGTTTTTACAGAAGAA AATG
Epyc	CAGCAGAACTCCTCAAGCCTA	GCATCTCAAATATACTGAGTT TGCT
Frzb	GCTGCCTCTGTCCTCCACT	CGATCCTTCCACTTCTCAGC
Ifit3	AGGCCATGGAGTTGAATCCT	AAACTGAGCTGCCTTTTGGA
Myo6	GGAGGCATCCAGTACCTTCA	AGGAAGTGGTGAGCCTTCG
Nkain4	CTCTGGAACGGCAAGTCTTTG	GTGGCCGGTATTGAATGGTG
Optn	TGGATGAGATGAAGCAGACG	CTGCTCTCTCAGCGTGAAAA
Prg2	CCCAAGGAAGAGGACACAAC	AACACTGAAACTGTGGATGGAG
Qpct	GCACACCATGGATGACAATG	TGGTGTCTTGAAGTTGCTGTAGA
Vldlr	AGGTGGCTACTTGATGTGGAG	ATTGCTGGGTATGTGTGTCCT

### Immunocytochemistry

Cochleae were perfused with 4% formaldehyde in phosphate buffered saline (PBS). The inner ear sensory epithelium was dissected out. The tissue was treated with 0.2% Triton X-100/PBS and goat serum (10%) was used to block non-specific binding. The tissue was then incubated with primary antibodies and washed with PBS, followed by incubation with secondary antibodies (Life Technologies, Lot# 1579044), followed by three washes in PBS. Primary antibodies used were rabbit polyclonal or monoclonal antibodies against p75NTR (NGFR: 1:300; Advanced Targeting Systems AB- N01 AP), LUZP2 (1:400, NBP2-58798, Novus), KCNJ16 (1:300, GTX54791, Gene Tex), and TUBB3 (1:400, MMS-435P, Covance). The samples were mounted on glass slides with antifade solution (Prolong Antifade Kit, Invitrogen, Carlsbad, CA) before imaging on a Leica Confocal Microscope (Leica TCS SP8 MP). Three cochleae from three adult mice were used for immunodetection of the expression for each gene/protein.

### Statistical analysis

Means and standard deviations from biological and technical repeats were calculated. ANOVA False Discovery Rate-corrected *p*-values were used to compare average expression (RPKM) values from four different cell populations for each transcript from repeats and *p* ≤ 0.01 was considered statistically significant.

## Results

### Gene expression profiles of pillar and deiters' cells

We detected 18,217 and 22,807 transcripts that had expression values greater than zero in pillars and Deiters' cells, respectively. With an arbitrary value of 0.1 RPKM as cutoff for background level expression, 15,602 and 16,005 transcripts were considered to be expressed in pillar and Deiters' cells, respectively, with 14,486 transcripts expressed in both populations. For IHCs and OHCs, 19,730 and 21,166 transcripts were detected. 15,653 and 16,364 transcripts were considered to be expressed. These numbers of transcripts in IHCs and OHCs are similar to those reported in our previous study using the DNA microarray technique (Liu et al., 2014). We compared transcriptomes among four different types of cells in the organ of Corti. Figure 1C presents a Venn diagram, depicting the number of expressed transcripts in each of the four cell types. The number indicates the total transcripts shared among two or more cell types or those uniquely expressed by a single cell type. As indicated, 13,272 transcripts are commonly expressed in all four cell types, although the number in common varies when comparison was made among different cell types. Uniquely expressed transcripts only account for 2.9 to 4.9 percent of the total transcripts expressed in one cell type when any two cell types were compared. There are 13,648 and 13,959 protein coding genes in pillar and Deiters' cells, respectively, accounting for 87.5 and 87.2% of the total transcripts expressed in these two cell types. In comparison, protein coding genes account for 84.8 and 83.2% of the transcripts expressed in IHCs and OHCs, respectively. Figure 1D shows the overall gene expression profiles of pillar cells and Deiters' cells using the expression levels of IHCs and OHCs as reference.

**Figure 2:**
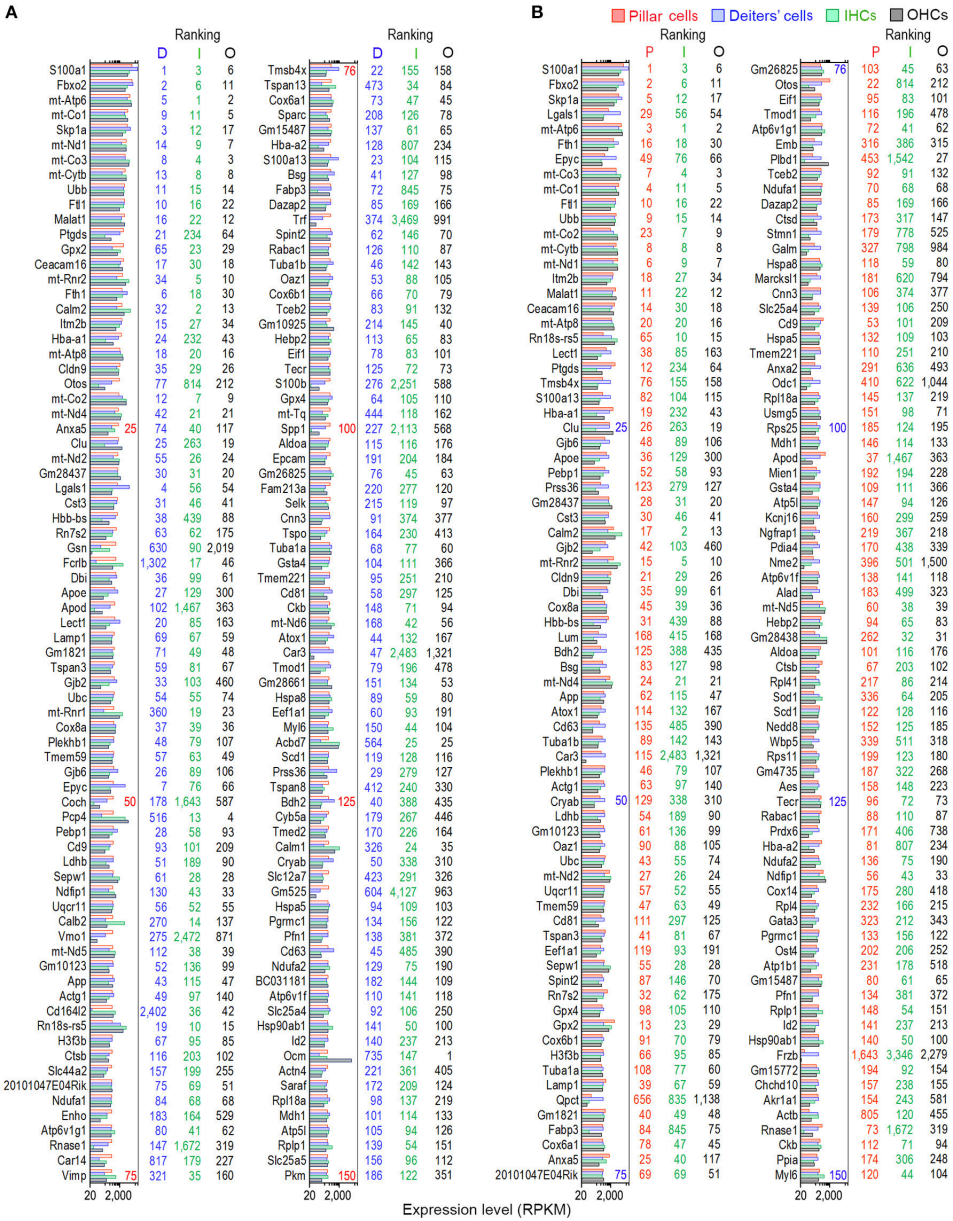
Expression levels of the top 150 transcripts in pillar **(A)** and Deiters' **(B)** cells in descending order. The expression values of the same transcripts in IHCs and OHCs are also presented. The color-coded numbers on the right side of each panel represent the ranking of the same transcript in the genes expressed in pillar (P), Deiters' (D), IHCs (I) and OHCs (O).

We examined the most abundantly expressed genes in pillar and Deiters' cells and compared the top 150 genes expressed in all four types. Figure 2A exhibits the expression levels for the 150 most abundant transcripts in pillar cells. Expression levels and abundance rankings for the same transcripts in Deiters, IHCs and OHCs are also illustrated for comparison. In the same way, the 150 most abundant transcripts in pillar cells compared to the same transcripts and abundant rankings in Deiters' cells, IHCs and OHCs are presented in Figure 2B. As it is apparent from the two figures, the majority of the transcripts abundantly expressed in one population are also richly expressed in other three cell types.

### Similarity analysis of gene expression profiles among different cell types

**Figure 3:**
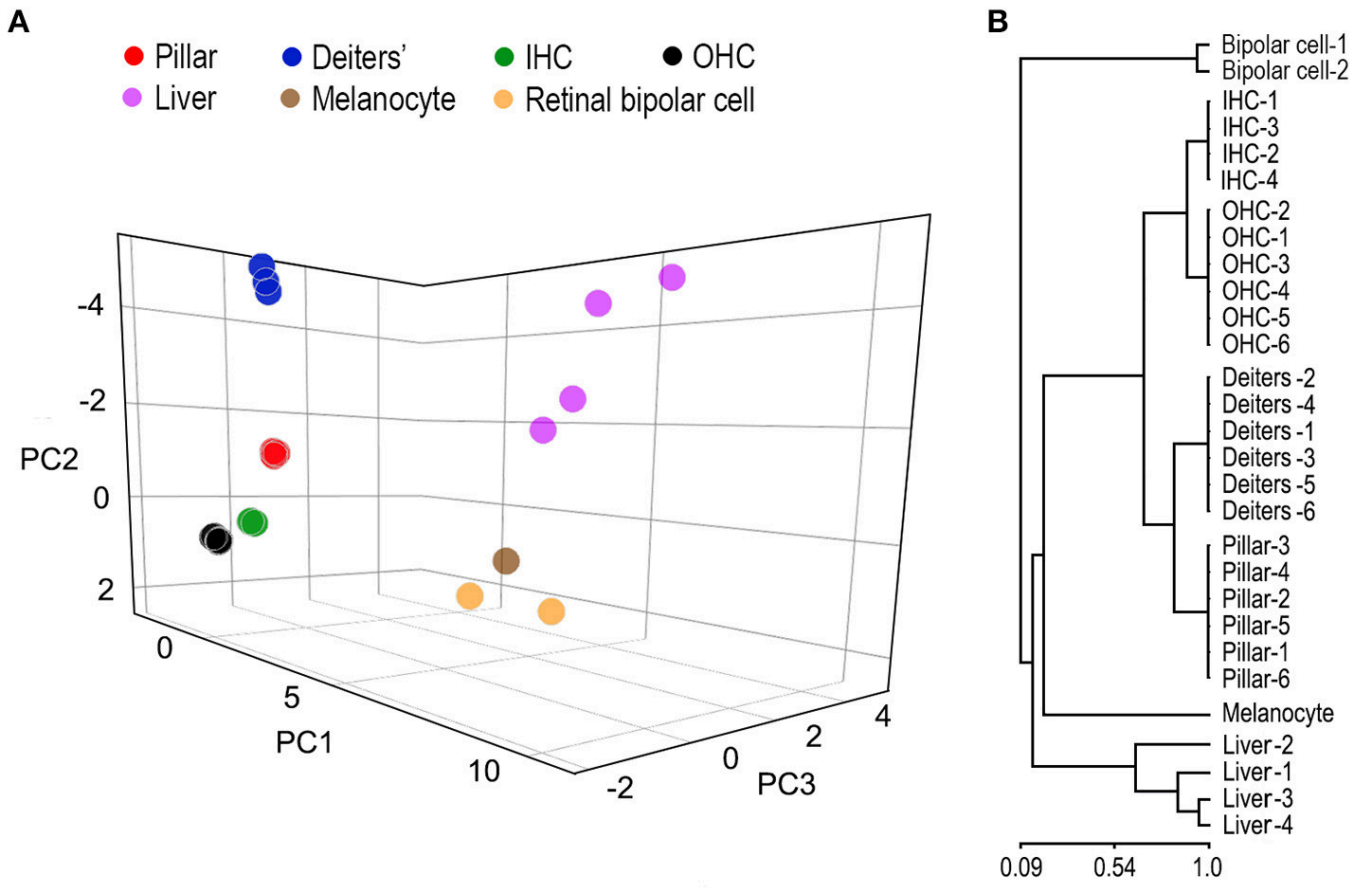
Similarity measures of gene expression profiles among different cell types. **(A)** Principal component analyses of the gene expression profiles of seven different cell types. RNA-seq-based gene expression data of liver cells and retinal bipolar cells were obtained from published work (Shekhar et al., 2016; Fradejas-Villar et al., 2017). Stria melanocyte dataset was from our own unpublished work. These data are normalized with datasets from the organ of Corti. **(B)** Hierarchal clustering analysis of seven cell types.

SCs and HCs in the organ of Corti are both derived from prosensory progenitor cells during development. We examined underlying biological relationships in gene expression profiles among four different cell types using gene hierarchical clustering analyses. RNA-seq-based transcriptomic data of stria melanocytes (our unpublished data), liver cells, and retinal bipolar cells from published studies (Shekhar et al., 2016; Fradejas-Villar et al., 2017) were also used for comparison. Figure 3A presents the similarity measure of gene expression profiles of the seven cell types using Principal Component Analysis or PCA (Yeung and Ruzzo, 2001). The distance measure shown in the three-dimensional plot reflects global similarity of the gene expression profiles among different cell types. As shown, gene expression profiles of biological replicates of the same cell types from the present study are highly reproducible. Not surprisingly, IHCs and OHCs are highly analogous compared to other cell types. Similarly, pillar and Deiters' cells are also analogous to each other; however, the gene expression profile of pillar cells is more similar to HCs than that of the Deiters' cells. Retinal bipolar cells and stria melanocytes, both of neuronal origin, are more closely related to each other but distinct from HCs. Liver cells are even more distinct, reflecting their significant difference from the other cell types, which all have a neural or sensory origin. Similarly, a hierarchical clustering analysis supports these findings (Figure 3B).

### Enriched genes in pillar and deiters' cells

**Figure 4:**
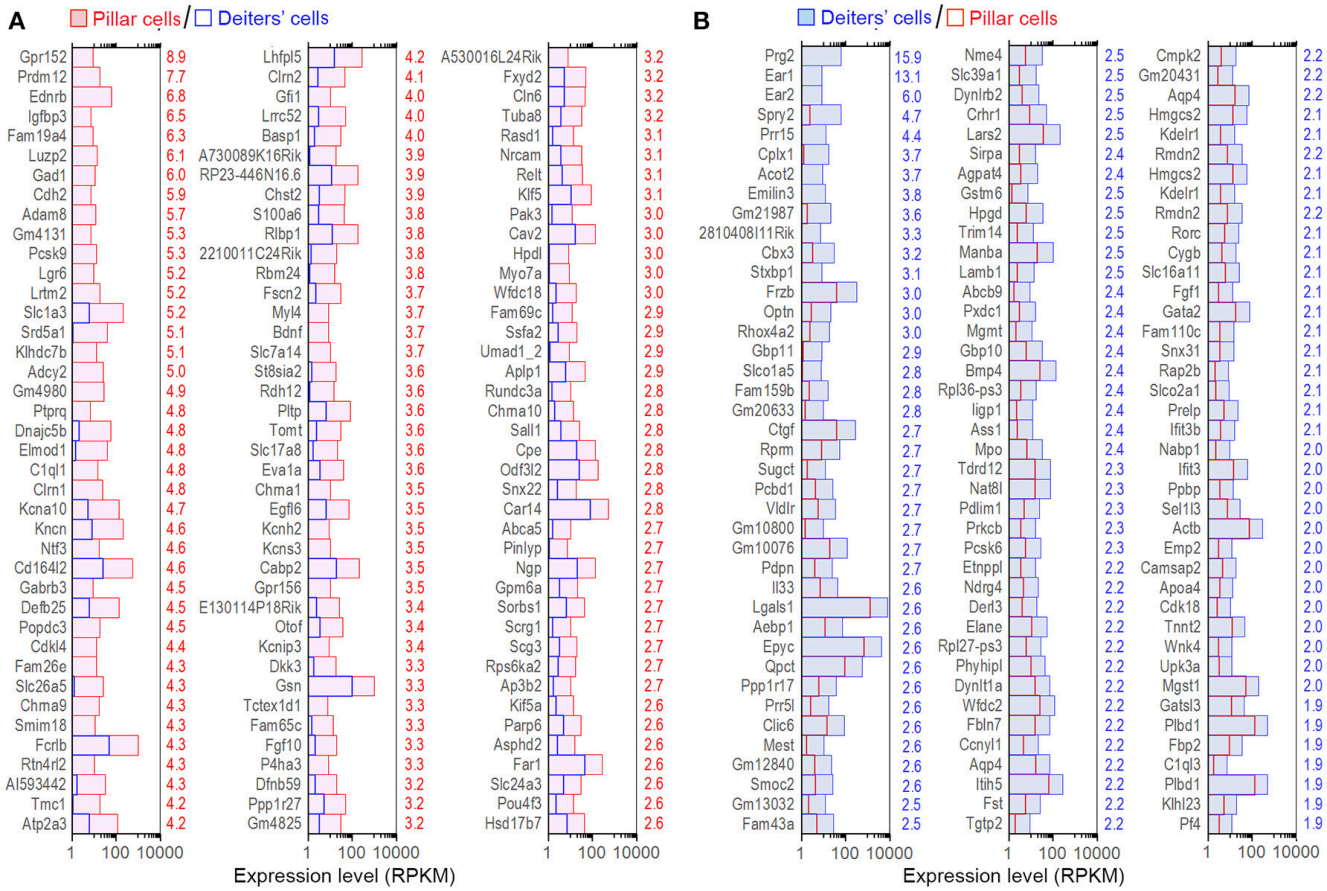
Top 120 enriched genes expressed in pillars and Deiters' cells. **(A)** Top 120 enriched genes expressed in pillar cells with reference to Deiters' cells. **(B)** Top 120 enriched genes expressed in Deiters' cells with reference to pillar cells. The numerical values (color-coded with red for pillar cells and blue for Deiters' cells) on the right side of each panel represent Log2-fold difference in expression between the two cell types.

Although the majority of the transcripts are commonly expressed in all four cell types, it is important to identify those that are enriched in each cell type as those transcripts may be responsible for unique biological properties. Enriched genes were defined as those whose expression levels were above background and at least 2-fold difference between the two cell types with statistical significance (with *p* ≤ 0.01). We first compared gene expression between pillar and Deiters' cells to identify enriched genes in either population. Such comparison identified 898 and 1,211 genes enriched in Deiters' and pillar cells, respectively. We also identified 574 and 429 uniquely expressed genes in Deiters' and pillar cells, respectively, among the four cell types examined from the organ of Corti. Figure 4 shows the expression levels and log2-fold difference (values on the right side of each panel) of the top 120 enriched transcripts in pillar and Deiters' cells when they were compared to each other. While the function of most genes shown in Figure 4 has not been characterized in SCs, it is apparent that pillar cells have a greater level of expression of the genes (such as Tmc1, Chrna9, Chrna10, Slc17a8, Otof, Slc26a5, Pou4f3, Slc1a3, Dnajc5b, Clrn1, and Kncn) that are known to be related to HC function and morphology.

**Figure 5:**
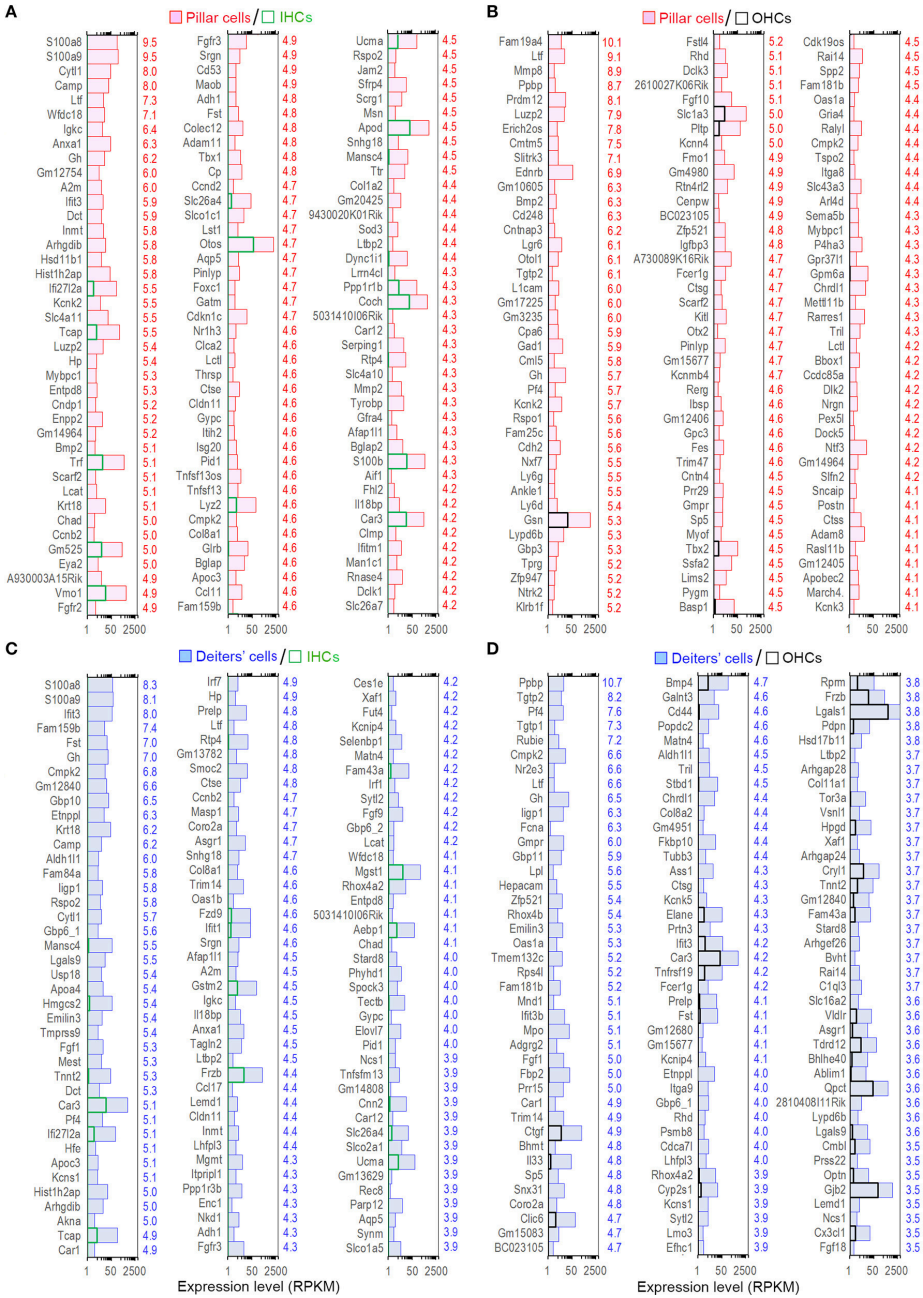
Top 120 enriched genes expressed in pillars and Deiters' cells. **(A,B)** Top 120 enriched genes expressed in pillar cells with reference to IHCs **(A)** and OHCs **(B)**. The values in red on the right side represent Log2-fold difference in expression for pillar cells vs. IHCs or OHCs. **(C,D)**: Top 120 enriched genes expressed in Deiters' cells with comparison to IHCs **(C)** and OHCs **(D)**. Fold difference (in blue) is presented on the right side of each panel.

The expression levels of the transcripts of pillar and Deiters' cells were compared with those of IHCs. Such comparison showed 1,507 and 1,955 enriched genes, respectively in pillar and Deiters' cells. Figures 5A,C present the expression levels and log2-fold differences of the top 120 enriched transcripts in pillar and Deiters' cells compared to IHCs. When compared with OHCs, pillar cells had 1,255 enriched genes and Deiters' cells had 1,184 enriched genes. Figures 5B,D show the top 120 enriched transcripts in pillar and Deiters' cells, compared to OHCs. In enrichment analyses, pillar and Deiters' cells shared more enriched genes than either OHCs or IHCs; however, Deiters' cells shared a greater number of enriched genes with OHCs, while pillar cells shared a greater number of enriched genes with IHCs.

**Figure 6:**
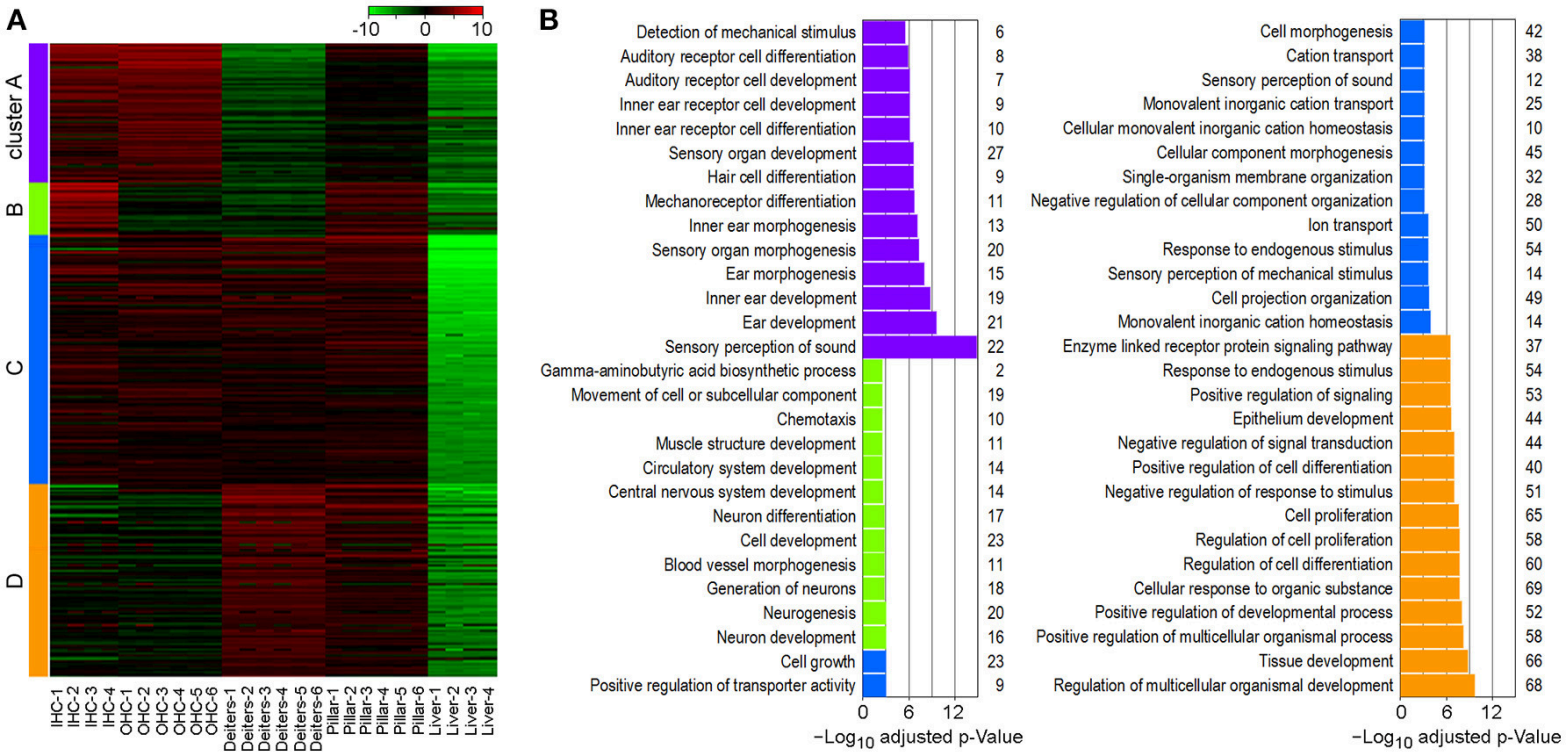
Gene enrichment analysis among inner ear cell types. **(A)** Heatmap generated using K-means clustering analysis of 1,200 genes into four clusters, based on enrichment of associated biological processes among the cell types. Liver cells were included as a negative control. **(B)** Significantly enriched biological processes corresponding to clustering analysis. The *x*-axis shows the negative log_10_ adjusted *p*-value and the number of genes in each enrichment category are shown on the right.

To determine gene enrichment corresponding to biological processes among the cell types we performed a k-means clustering analysis using iDEP. A total of 1,200 genes were clustered into four associated groups based on similar expression patterns among the cell types (Figure 6). Cluster A (*N* = 310) showed significant enrichment in biological processes, such as sensory perception of sound, inner ear development and HC differentiation. These processes are highly upregulated in IHCs and OHCs; however, pillar cells also showed increased expression of these genes, further supporting a higher degree of relatedness among these cell types. Shared enrichment among IHCs and pillar cells was also noted in Cluster B (*N* = 105), which is associated with biological processes, such as neurogenesis. All four inner ear cell types had significant enrichment in Cluster C (*N* = 428) which included many biological processes associated with general HC and sensory cells functions including cell projection organization and ion transport. Lastly, both Deiters' and pillar cells had upregulated genes in Cluster D (*N* = 357), which includes biological processes involving regulation of tissue development, cell proliferation, and cell response. Note that almost all of the genes in each cluster are downregulated in liver cells, while the commonly upregulated genes further support the enriched biological functions among the cell types of the inner ear.

### Pillar and deiters' cells express genes for HC specialization machinery

**Figure 7:**
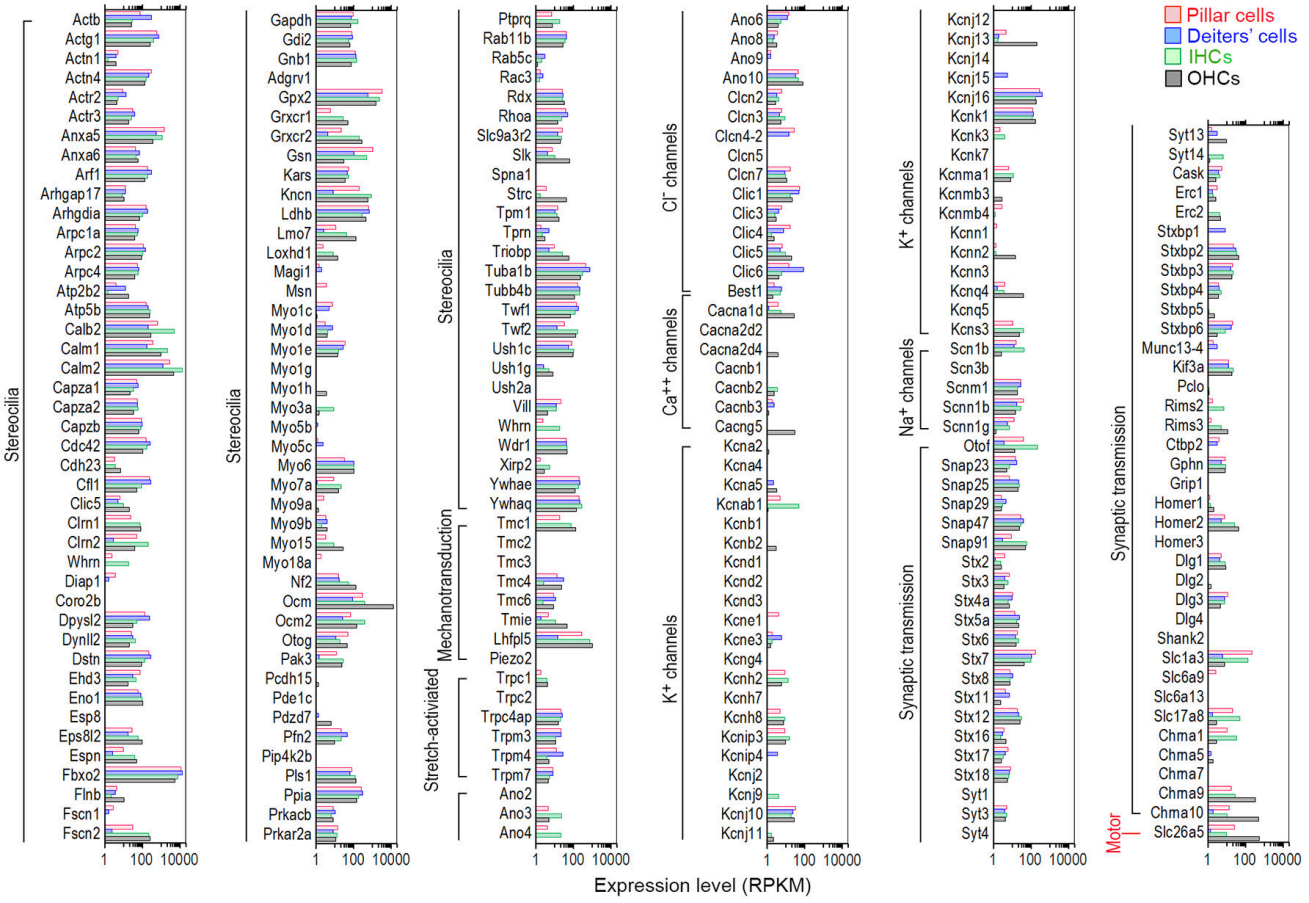
Comparison of expression levels of genes related to HC specializations among pillar cells, Deiters' cells, IHCs, and OHCs. Genes that are currently known to encode proteins important for mechanotransduction apparatus (stereocilia structure, tip-links, and mechanotransduction channels), ion channels, synaptic transmissions, and somatic motility in the apical, basolateral and synaptic membranes are included.

HCs contain mechanotransduction apparatus in the stereocilia bundle in the apical surface, and ion channels and pre- and post-synaptic specializations in the basolateral and synaptic membranes. OHCs also possess the motor protein prestin (SLC26A5, Zheng et al., 2000) in the basolateral membrane. These morphological and functional specializations are largely responsible for HCs to function as sensory receptor cells. We utilized categories based on HGNC Gene Families/Groupings Nomenclature to analyze genes encoding proteins related to these specializations. Recent mass spectrometry studies also identified >1,100 proteins in stereocilia bundles of mouse vestibular HCs (Krey et al., 2015; Wilmarth et al., 2015). Figure 7 shows expression levels for 102 transcripts encoding stereocilia-associated proteins in IHCs and OHCs. It is apparent that most of those genes expressed in HCs are also expressed in pillar and Deiters' cells. Myo6, considered as a HC-specific marker, is expressed in Deiters' cells (RPKM value: 109.22) with the expression level comparable to IHCs (99.36) and OHCs (102.7). The expression of Myo6 is also detected in pillar cells (33.76). Myo7a, another HC-specific marker, is also expressed in pillar and Deiters' cells, and Myo15 is detected in pillar cells.

All HCs contain mechanotransduction channels in the stereocilia. Five proteins (TMHS/LHFPL5, TMIE, TMC1, TMC2, and PIEZO2) are candidates of mechanotransduction channels and accessory elements (Kawashima et al., 2011; Pan et al., 2013; Wu et al., 2017). Tmc1, expressed at relatively high levels in both IHCs (61.16) and OHCs (127.27), was expressed in pillar (19.34) and Deiters' (0.91) cells (Figure 7). Tmc2 was not detected in any of the cell types in adult mice. The roles of Tmc4 and Tmc6 are unknown, but they were also expressed in all four cell types. Lhfpl5, a component of the HC's mechanotransduction machinery that may couple PCDH15 to the transduction channel (Xiong et al., 2012), was highly expressed in IHCs (637.73) and OHCs (1061.74). It was also expressed in Deiters' cells (12.12), and especially in pillar cells (270.92) with relatively high level of expression. TMIE may form a ternary complex with the tip-link component PCDH15 and its binding partner TMHS/LHFPL5 (Zhao et al., 2014). Tmie was expressed in all four cell types, with highest expression in OHCs (48.23). Interestingly, Piezo2, encoding PIEZO2 protein that may be the second ion channels in the apical surface of HCs (Wu et al., 2017), was not detected in any of the four cell types. Piezo2 was also not detected in our microarray study (Liu et al., 2014). We also examined genes encoding stretch-activated ion channels in HCs, although that these channels are unlikely responsible for mechanotransduction in the stereocilia (Wu et al., 2016). Moderate expression of transient receptor ion channel genes Trpc4ap, Trpm3, Trpm4, and Trpm7 was detected in all four cell types. Relatively low-level expression of Cdh23 and Pcdh15, encoding tip-link components, was also detected in pillar and Deiters' cells.

Ion channels in the basolateral and synaptic membranes of HCs are important for establishing and maintaining membrane potential, shaping receptor potentials, trigging neurotransmitter release, and regulating cell volume (Kros, 1996). Figure 7 lists genes that encode various types of Cl^−^, Ca^2+^, Na^+^, and K^+^ channels. Most ion channel-related genes that are expressed in HCs were also expressed in pillar and Deiters' cells. However, several genes had greater expression in pillar and Deiters' cells than in HCs. For example, the expression of Cl^−^ channels genes (Ano6, Clic1, Clic3, Clic4, and Clic6) was greater in pillar and Deiters' cells than in HCs. Kcnj16, encoding an inward-rectifier type potassium channel that may be involved in the regulation of fluid and pH balance, was expressed in all four cell types with greater expression in pillar and Deiters' cells. Kcnj10 and Kcnk1 were moderately expressed in these four cell types. Genes encoding calcium channels (Cacna1d, Cacnb3, and Cacng5) were also expressed in all four cell types, although their expression in pillar and Deiters' cells was at lower levels.

HCs contain ribbon synapses that are associated with afferent innervation. HCs, especially OHCs, also contain efferent synapses with nicotinic cholinergic receptors alpha9/10 subunits (Elgoyhen et al., 1994, 2001). We examined genes associated with neurotransmitter vesicle transport and release, pre- and post-synaptic scaffolding proteins, and nicotinic cholinergic receptors in pillar and Deiters' cells. It is apparent from Figure 7 that many genes associated with synaptic transmission are expressed in pillar and Deiters' cells at levels similar to HCs. Otof was highly expressed in IHCs (Yasunaga et al., 1999), followed by pillar cells, OHCs and Deiters' cells. Ctbp2 (Ribeye) was also expressed in pillar and Deiters' cells. Slc17a8 (Ruel et al., 2008), encoding VGLUT3 that transports glutamate into synaptic vesicles, was also expressed in pillar cells with an expression level greater than that of OHCs. Interestingly, Slc1a3, encoding the GLAST protein (a member of a high affinity glutamate transporter family) and necessary for rapidly removing released glutamate from the synaptic cleft (Velaz-Faircloth et al., 1996), was detected in both IHCs and OHCs with differential expression favoring IHCs. Slc1a3 was also detected in adult zebrafish HCs and mouse cochlear and vestibular HCs using RNA-seq (Scheffer et al., 2015; Barta et al., 2018) and microarray (Liu et al., 2014). Previous studies using electrophysiology, immunocytochemistry and electron microscopy have shown that GLAST is mainly detected in SCs surrounding IHCs (Furness and Lehre, 1997; Glowatzki et al., 2006) and in vestibular HCs (Takumia et al., 1997). Figure 7 shows that the highest expression of Slc1a3 is seen in pillar cells, followed by IHCs. Chrna10 and Chrna9 were also detected in pillar and Deiters' cells with levels comparable to IHCs. Finally, Slc26a5, encoding the motor protein prestin of OHCs (Zheng et al., 2000), was highly expressed in OHCs followed by pillar cells. Lower level expression of Slc26a5 was detected in Deiters' cells and IHCs. Slc26a5 is known to have five alternative spliced transcripts, three of which are protein coding genes. Interestingly, 4 transcripts were detected in both OHCs and pillar cells. Two transcripts were expressed in IHCs and one was expressed in Deiters' cells. Taken together, comparisons between pillar cells and Deiters' cells showed that pillar cells have a higher level of expression of genes that are related to HC functions including mechanotransduction (such as Tmc1, Lhfpl5, Tmie and Cdh23), basolateral membrane conductance (Cacna1d, Cacng5, Kcnj13, Kcnj16), synaptic transmission (such as Slc17a8, Otof, Slc1a3, Chrna9, and Chrna10), and OHC electromotility (Slc26a5).

### Expression of cell cycle control and deafness-related genes in pillar and deiters' cells

**Figure 8:**
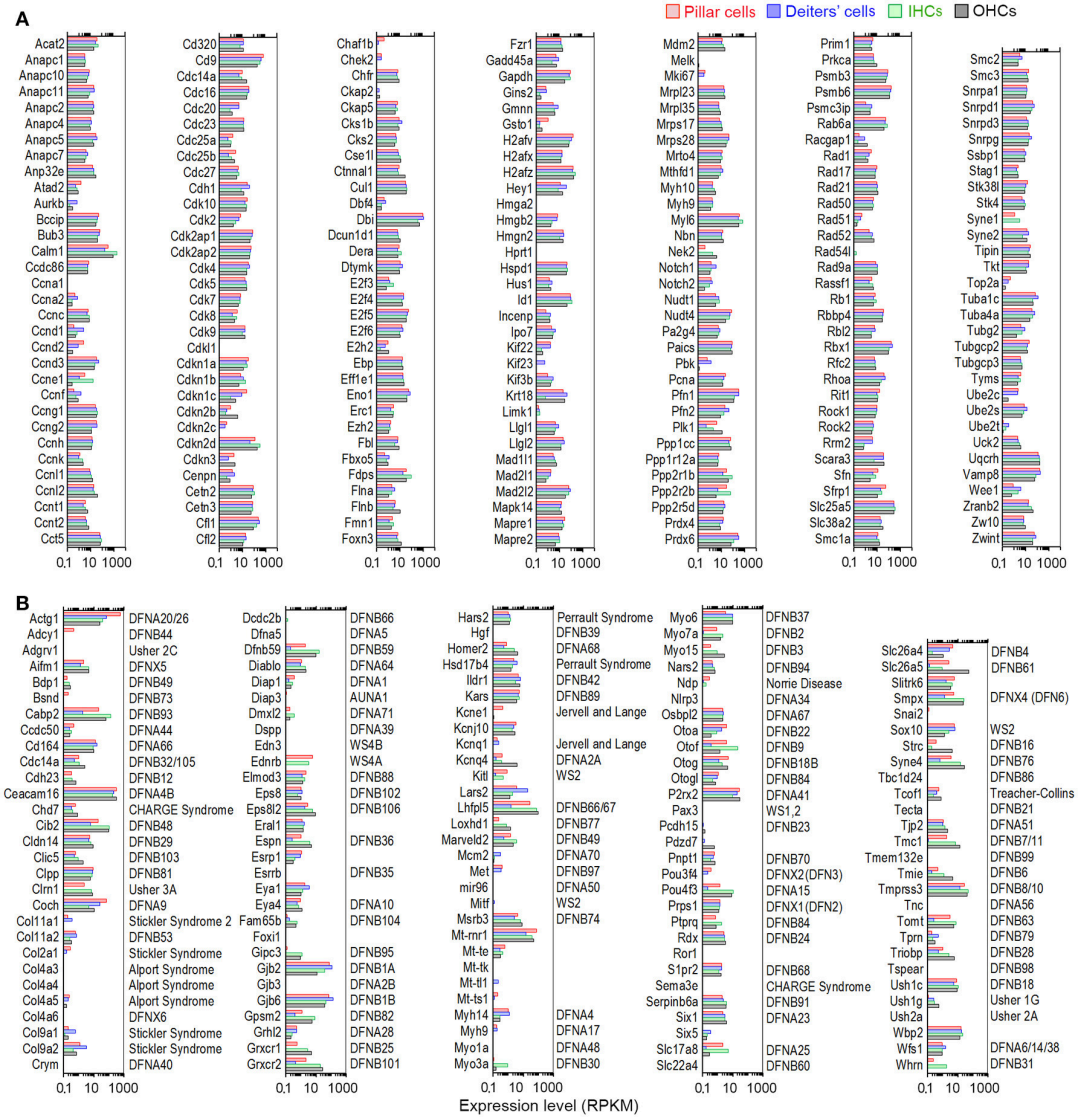
Genes regulating cell cycle and genes associated with hereditary deafness. **(A)** Expression levels of genes that are known to be important for cell cycle regulation in pillar cells, Deiters' cells, IHCs, and OHCs. **(B)** Comparison of expression levels of 101 known deafness-associated genes in pillar cells, Deiters' cells, IHCs, and OHCs.

We also analyzed expression of genes important for cell cycle regulation in pillar and Deiters' cells. Analyses of cell cycle regulation genes in these two cell types may identify differences between mammalian and lower vertebrate SCs and illuminate a road map for targeted manipulation of genes to induce SC conversion to HCs in mammals. It is estimated that the mammalian cells may have up to 1,000 genes that are involved in cell cycle regulation (Forrest et al., 2003). We examined the expression of 223 genes commonly assayed in a PCR array for cell cycle regulation. These genes are involved in promoting or inhibiting progression of the cell cycle and regulating the transitions between each of the phases, as well as DNA replication, checkpoints, and arrest. Except for a few genes that were not detected, the majority were expressed in pillar and Deiters' cells with the levels similar to HCs (Figure 8A).

Mutations or deficiencies affecting ~144 genes have been linked to inherited syndromic or non-syndromic hearing loss (Smith et al., 2014). We examined the expression of these genes to determine which genes are expressed in each cell type. Figure 8B presents expression levels of these genes in pillar, Deiters' cells, IHCs and OHCs. Seven genes (Hgf, Gjb3, Mir96, Col4a6, Foxi1, Myo1a, and Pax3) were either not detected or below the cutoff level in all four cell types, while five genes (Tecta, Esrrb, Diap3, Col4a3, and Edn3) were only detected in one or two cell types with near cutoff level of expression. The majority of the genes that were expressed in HCs were also expressed in pillar and Deiters' cells, although most of them were differentially expressed.

### Verification by q-PCR and immunodetection

**Figure 9:**
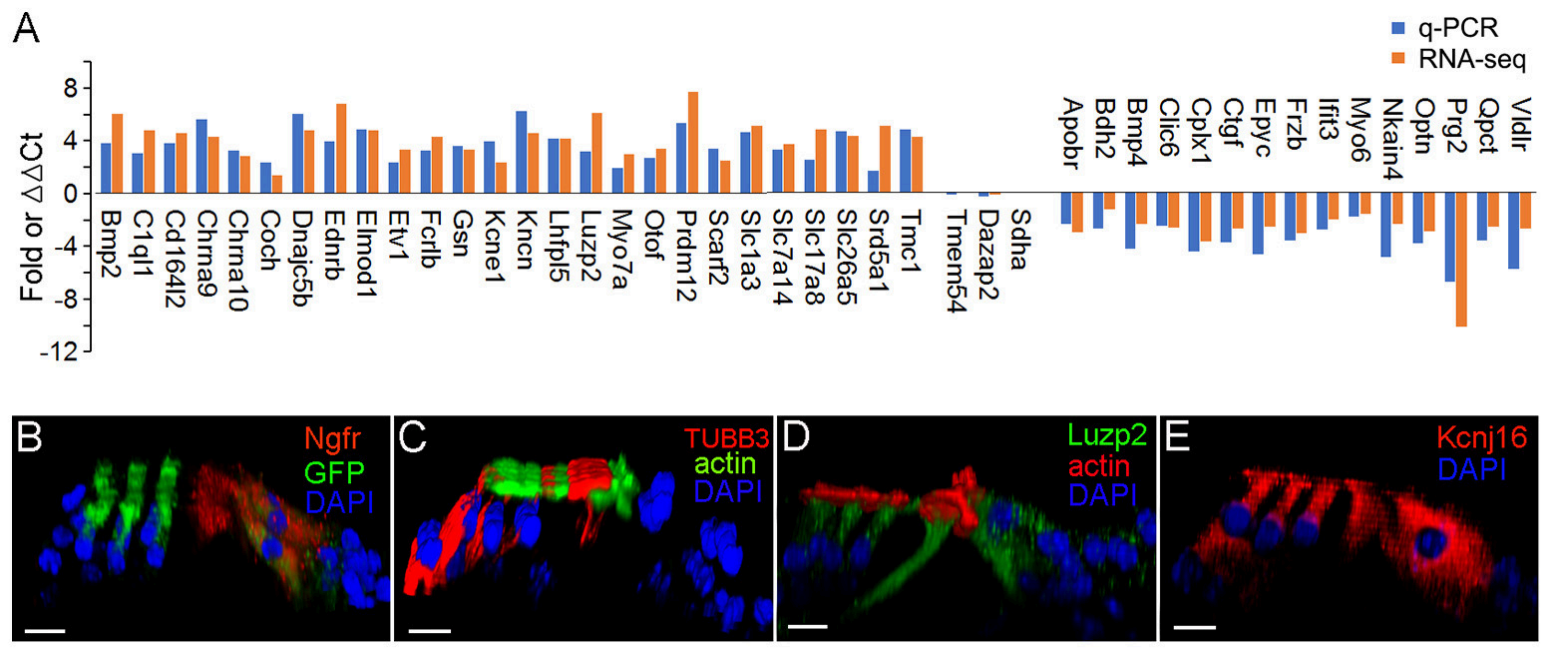
Validation of expression of representative genes in pillar and Deiters' cells using q-PCR and immunocytochemistry. **(A)** Fold differences in expression of 41 genes between pillar and Deiters' cells using q-PCR and RNA-seq. Positive values indicate higher gene expression in pillar cells than in Deiters' cells while negative values indicate higher expression in Deiters' cells than in pillar cells. Fold differences were calculated in log2 base for RNA-seq data while ΔΔCt values were used for q-PCR. **(B–E)**: Images of confocal optical sectioning of the organ of Corti stained with 4 different antibodies against NGFR **(B)**, TUBB3 **(C)**, LUZP2 **(D)**, and KCNJ16 **(E)**. In **(B)**, *Atoh1*^tm4.1Hzo^/J mice were used and thus, HCs expressed GFP. Bars in all images represent 10 μm.

We used q-PCR to validate the expression of 41 representative genes that are differentially expressed either in pillar or Deiters' cells. Fifteen additional mice were used to prepare three biological replicates of pillar and Deiters' cells for qPCR validation. The gene expression profiles of IHCs and OHCs were highly consistent with our microarray study (Liu et al., 2014). The HC-related genes detected in RNA-seq (Figure 7) were all detected in microarray analysis (Figure 6B in Liu et al., 2014). More importantly, the differential expression patterns of those genes between IHCs and OHCs are highly consistent between the two different platforms. Among 368 genes that displayed a differential expression pattern between IHCs and OHCs in the microarray analysis (Figure 4 of Liu et al., 2014), not a single gene displayed a different pattern in the RNA-seq datasets. In addition, the genes that are differentially expressed in either IHCs and OHCs are also highly consistent with virtually all previous studies using immunocytochemistry or *in situ* hybridization. For these reasons, we focused our verification on pillar and Deiters' cells. Three genes that showed no significant difference in the level of expression between pillar and Deiters' cells in RNA-seq analysis were also examined for validation. Many of the genes used for validation are known to be related to HC function and structure. Figure 9A shows the differential expression of 44 genes in pillar and Deiters' cells using q-PCR. For comparison, the RPKM values from RNA-seq are also presented. As shown, three genes (including Sdha) that have similar level of expression (with no statistical significance, *p* ≥ 0.05, *n* = 3) between pillar and Deiters' cells in RNA-seq analysis do not show any significant difference (*p* ≥ 0.05, *n* = 3) in q-PCR analysis as well. However, the genes that are differentially expressed in either pillar or Deiters' cells in RNA-seq analysis also show differential expression in q-PCR analysis. As it is apparent from Figure 9A, the differential expression patterns (reflected by change in the same direction in the plot) of 41 genes in pillar or Deiters' cells are highly consistent between the two techniques, although the fold difference values vary.

We examined the expression of four genes using antibody-based immunocytochemistry and confocal microscopy. Ngfr (p95) is mainly detected in pillar cells during development. Figure 9B shows that it is also detected in adult pillar cells. The expression of Tubb3, Luzp2 and Kcnj16 in any of these four cell types has not be reported before. Figure 9C shows that TUBB3 was detected in Deiters cells and weakly detected in pillar cells. As shown in Figure 9D, strongest expression of LUZP2 is seen in pillar cells. LUZP2 is also detected in OHCs and IHCs. Luzp2 has the highest expression in pillar cells in RNA-seq analysis, consistent with our immunostaining study. RNA-seq analysis showed that Kcnj16, encoding potassium voltage-gated channel subfamily J member 16, is expressed in all four cell types. This is consistent with the expression pattern of KCNJ16 seen in Figure 9E. Mutation of this gene is known to cause SeSAME syndrome, which is characterized by seizures, sensorineural deafness and ataxia.

## Discussion

This study represents the first genome-wide transcriptome analysis that examines gene expression profiles of highly purified and pristine IHCs, OHCs, pillar, and Deiters' cells. The cell-specific transcriptomes provide important information about the molecular mechanisms underlying biological properties of these cells, which will be critical for HC regeneration in adult mammals. This analysis has also established a road map for future characterization of genes expressed in these four cell types. Several recent studies have examined transcriptomes of SCs and HCs from embryonic and neonatal mice (Burns et al., 2015; Waldhaus et al., 2015; Maass et al., 2016; Cheng et al., 2017). While the study by Waldhaus et al. (2015) provided a quantitative spatial map of the gene expression profiles of different cell types along the longitudinal and radial axes of the organ of Corti in neonatal mice, other studies examined gene expression profiles of SCs during development (Burns et al., 2015) and in response to inhibition of Notch signaling (Maass et al., 2016; Cheng et al., 2017). In contrast to previous studies analyzing SCs and HCs during development, the present study is focused on gene expression profiles of adult SCs and HCs. This is important as the gene expression profiles of immature and mature SCs and HCs are different. Furthermore, SCs and HCs in the present study were identified and individually collected based on their distinct morphology, thus minimizing contamination. Our transcriptomic analyses showed that 81 to 85 percent of the transcripts are commonly expressed in all four adult cell types. This may reflect the fact that HCs and SCs both originate from prosensory progenitor cells during cochlear development. More importantly, we revealed that adult pillar cells and Deiters' cells also express the genes that encode machinery for HC specializations, providing strong molecular evidence that these two cell types can be targeted for conversion to HCs in adult mammals.

Although a significant majority of transcripts are commonly expressed among pillar cells, Deiters' cells and HCs, we detected several differentially expressed genes in pillar and Deiters' cells. The function of many of these genes have yet to be characterized. Several genes are particularly interesting, as we speculate that these genes may be related with unique function and morphology of pillar and Deiters' cells. These genes include: S100a8, S100a9, Cytl1, Camp, Ltf, Wfdc18, Fam19a4, Mmp8, Rrdm12, Ppbp, Fst, Lfit3, Tgtp2, Pf4, Gpr152, Prdm12, Ednrb, Prg2, Ear1, Ear2, and Spry2. These genes represent excellent targets for examining gene-specific roles in pillar and Deiters' cells using loss-of-function approaches. We also showed that many of the known deafness-related genes are expressed in pillar and Deiters' cells (Figure 8B). Knowing which genes are expressed in different cell types will provide a better understanding of the molecular mechanisms of hearing loss in humans.

Specializations in the apical, basolateral and synaptic membranes confer HCs with unique morphology and function. Based on the high similarity of the transcriptomes between SCs and HCs, we predicted a sharing of these specialized genes. Two groups of genes related to mechanotransduction and synaptic transmission are particularly important since they are critical for HCs to function as an auditory receptor cell. Mechanotransduction is mediated by a mechanotransduction apparatus in the stereocilia. Tip links (Pickles et al., 1984), mechanotransduction channels, and myosin motor are collectively regarded as the mechanotransduction apparatus. Studies have shown that cadherin 23 and protocadherin 15 interact to form tip-link filaments (Kazmierczak et al., 2007) while TMC1 is a candidate for the mechanotransduction channel (Kawashima et al., 2011; Pan et al., 2013). Myosin1c, linking the transduction channel to the actin core of the stereocilia, may adjust the tension of the tip links and mediate slow adaptation of the mechanotransduction (Gillespie and Corey, 1997; Holt et al., 2002). We detected moderate expression of those genes (Cdh23, Pcdh15, Tmc1, and Myo1c) cognate the transduction apparatus in pillar and Deiters' cells. Pillar and Deiters' cells should also express genes related to synaptic machinery if they have the potential to function as HCs. We showed that pillar and Deiters' cells have moderate expression of synaptic transmission-related genes (Figure 7), such as those encoding the vesicular glutamate transporter (Slc17a8 or Vglut3), active zone proteins (Cask, Ctbp2, Erc1, Erc2, Pclo, Rims2, Rims3, and Unc13d), SNARE proteins (Snap23, Snap25, Snap47, Snap91, Stx7, Stx12, and Syt3), synaptogenesis proteins (Dlg1 and Dlg3), and calcium channels (Cacna1d, Cacnb3, and Cacng5). Genes encoding post-synaptic receptors (such as Chrna9 and Chrna10) and scaffolding proteins (such as Homer1, Homer2, Homer3, and Shank2) were also detected in pillar and Deiters' cells. Detection of genes encoding post-synaptic proteins and receptors in Deiters' cells suggests a molecular basis to support previous morphological and electrophysiological studies. These studies provided evidence that efferent fibers make contact with Deiters' cells (Wright and Preston, 1976; Nadol and Burgess, 1994; Burgess et al., 1997; Fechner et al., 1998) and that ACh application to Deiters' cells could evoke inward current (Matsunobu et al., 2001). Additionally, genes encoding ion channels shown to be expressed in HCs by previous molecular biology and electrophysiology studies were also detected in Deiters' cells and even more so in pillar cells. Furthermore, we also detected the expression of Kcnj10, Kcnj13, Kcnj16, and Kcnk1, whose function in HCs and SCs has not been characterized. Deiters' cells and pillar cells also express a lower level of Slc26a5, which encodes the motor protein of OHCs. Taken together, the expression of these important genes related to HC function and morphology strongly suggests that adult pillar and Deiters' cells have the intrinsic capacity to function as HCs.

Pillar cells are a newly derived cell type in mammals (Slepecky, 1996; Manley and Köppl, 1998). Based on the shared microtubular and actin filament cytoskeletal features and role of ion homeostasis maintenance, pillar and Deiters' cells were expected to have similar transcriptomes. The current study showed that although pillar and Deiters' have similar transcriptomes, pillar cells were found to be more related to HCs; whereas, Deiters' cells were relatively distinct. This is based on overall gene expression profiles and genes related to HC specialization machinery. Developmental studies show that nascent pillar cells are more likely to grow stereocilia bundles than Deiters' cells when Notch signaling was blocked (Li et al., 2018). Furthermore, Notch inhibition or deletion of retinoblastoma family Rbl2/p130 also led to the generation of additional HCs in the IHC and pillar cell regions (Lanford et al., 1999; Rocha-Sanchez et al., 2011). A study using lineage trace in neonatal cochleae showed that new HCs, predominantly OHCs, arise from Lgr5-positive inner pillar and third row Deiters' cells (Bramhall et al., 2014). Our transcriptome analysis provides a molecular basis to support these observations. The analysis also suggests that pillar cells have a greater intrinsic capacity to be converted to HCs. We should point out that it is highly unlikely that contamination by HCs led to the finding that pillar cells are more similar to HCs than Deiters' cells, as the distinction between HCs and pillar cells is so striking at the light microscopical level.

While the genes encoding HC machinery are still expressed in adult SCs, such as the pillar and Deiters' cells, the key to conversion to HCs is to find the appropriate molecular trigger(s) and/or the mechanisms inhibiting their conversion. Future focus should be on the transcription factors (Lemon and Tjian, 2000), since the gene expression pattern in each cell type is regulated by transcription factors. The essential roles of transcription factors to reprogram mouse embryonic stem toward HC-like cells have been demonstrated (Oshima et al., 2010). A recent study shows that increased expression of Sox4 and Sox11 led to SC proliferation and new HC production in adult vestibular sensory epithelia (Gnedeva and Hudspeth, 2015). A list of HC-specific transcription factors has been compiled (Li et al., 2016). While numerous studies have shown that over-expression of Atoh1 and/or inhibition of Notch signaling can convert neonatal SCs to HC-like cells, the key(s) for spontaneous transdifferentiation of cochlear SCs have yet to be identified. Our transcriptomic analyses showed that pillar cells and Deiters' cells express the genes responsible for HC specializations in the apical, basolateral, and synaptic membranes that underpin the morphology and function of HCs in adult mammals, therefore, providing strong molecular evidence that these cells have the potential to be converted to HCs.

## Ethics statement

This study was carried out in accordance with the approved guidelines of the NIH. The protocol was approved by the Institutional Animal Care and Use Committee of Creighton University.

## Author contributions

HL, LC, and YL carried out the experiments. KG, SS, PJ, KB, and DH analyzed the data. DH designed the experiments and wrote the manuscript.

### Conflict of interest statement

The authors declare that the research was conducted in the absence of any commercial or financial relationships that could be construed as a potential conflict of interest.

## Supplementary material

Supplementary Video 1 Video showing an isolated OHC being drawn into the suction pipette.

Supplementary Table 1 A spreadsheet (in Excel format) showing gene expression values (in RPKM) of pillar cells, Deiters' cells, IHCs and OHCs from RNA-seq analysis. Values from each biological and technical replicate are all included. Normalized RPKM expression values of mouse stria melanocytes (our own, unpublished), retinal bipolar cells, and liver cells from previous RNA-seq-based studies are also included for comparison.
